# Alpha and beta adrenergic receptors modulate keratinocyte migration

**DOI:** 10.1371/journal.pone.0253139

**Published:** 2021-07-02

**Authors:** Hsin-ya Yang, Pieter Steenhuis, Aaron M. Glucksman, Zhanna Gurenko, Thi Dinh La, R. Rivkah Isseroff

**Affiliations:** 1 Department of Dermatology, University of California, Davis, Davis, California, United States of America; 2 Dermatology Section, VA Northern California Health Care System, Mather, California, United States of America; Emory University, UNITED STATES

## Abstract

Keratinocyte migration into skin wounds is the step of the healing process that correlates with the wound closure rate. Keratinocyte migration, and wound epithelialization are decreased when beta 2-adrenergic receptors (B2AR) are activated by 1 μM epinephrine/adrenaline, resulting in delayed wound healing in human and mouse skin. In the present study, we found paradoxically, that in a subset of keratinocyte strains exposure to low concentrations of epinephrine (0.1 nM) increased, rather than decreased, their migratory rate. We find that both the alpha- and the beta-adrenergic receptors are expressed in human keratinocytes, and expression of alpha-2 AR subtypes demonstrated for the first time. Therefore, we tested if the alpha-AR could be modulating the increased migratory response observed in these cell strains. By using specific inhibitors to alpha-AR, we demonstrated that blocking A2B-AR could reverse the rapid cell migration induced by the 0.1 nM epinephrine. Phosphorylation of ERK was elevated after 1–10 minutes of the low epinephrine treatment and the A2B-AR inhibitor blocked the ERK phosphorylation. The results suggest that both the A2B-AR and B2AR mediate keratinocyte migration, in which with a low level of epinephrine treatment, A2B-AR could alter the B2AR signals and regulate the migration rate.

## Introduction

After wounding, processes of inflammation, tissue formation and remodeling, and wound re-epithelialization sequentially establish adequate healing [1, 2]. From our past studies, the G-protein coupled beta adrenergic adrenergic receptor (AR) mediates important wound healing processes including keratinocyte migration and wound epithelialization [3–6], alteration of fibroblast reparative phenotype [7, 8], sustaining an inflammatory environment within the wound by increasing neutrophil dwell times [9], and increasing inflammatory cytokines within the wound [9]. One of the endogenous ligands for ARs is the stress hormone, epinephrine/adrenaline. Because the beta 2AR (B2AR) is expressed in most skin resident cells, and the ligands epinephrine and norepinephrine are generated by skin upon wounding tissue [6], we and others have examined the effects of B2AR activation on keratinocyte migration, an important component of skin wound repair. When an individual is under stress, supra-physiological (50 nM—1 μM) concentrations of epinephrine are present in the circulation and these supraphysiological levels inhibit keratinocyte migration *in vitro*, and impairing healing *in vitro*, *ex vivo* and *in vivo* [3–6, 10]. Blocking the binding of the ligand to the beta AR by administration of beta AR antagonist, such as timolol, reverses the inhibition and improves healing in pre-clinical animal models [5, 11–13]. Indeed, beta AR blockade has been proposed as a therapeutic for non-healing wounds [14], and venous or diabetic ulcers [15–17].

However, the range of physiologic concentrations epinephrine in human serum is wide: as low as 0.06 nM during sleep, and 0.11 nM while awake [18], 0.2 nM in hospitalized, resting patients [19, 20], and rising to 8.2 nM during exercise [21], and as high as 56 nM during cardiac arrest [19]. Here we queried the response of keratinocytes to the lower end of the physiologic range of serum epinephrine concentration (0.1 nM) and found that keratinocyte strains from different individuals exhibited an increase, rather than the previously observed decrease, in migratory speed noted in cells exposed to higher concentrations of epinephrine (physiologic at 10 nM, stress at 50 nM or supra-physiologic at 1 μM) [5, 19, 22].

This type of biphasic response to AR stimulation has been reported in mouse (MC4-L5) and human (IBH-4, IBH-6 and MDA-MB-231) breast cancer cells, with a mitogenic response to 10^−10^ M epinephrine, but inhibition of growth at higher concentrations (>10^−8^ M) [20]. Co-treating the cells with low dose epinephrine and rauwolscine, an inhibitor of the alpha-2C adrenergic receptor, abolished the increase in proliferation induced by epinephrine alone [20], suggesting both the A2AR and B2AR regulate the cell proliferation response. Here we tested the hypothesis that low, physiologic concentrations (0.1 nM) of epinephrine can activate both the A2AR and the B2AR, resulting in increased, rather than decreased, migratory speed.

## Materials and methods

**Fig 1:**
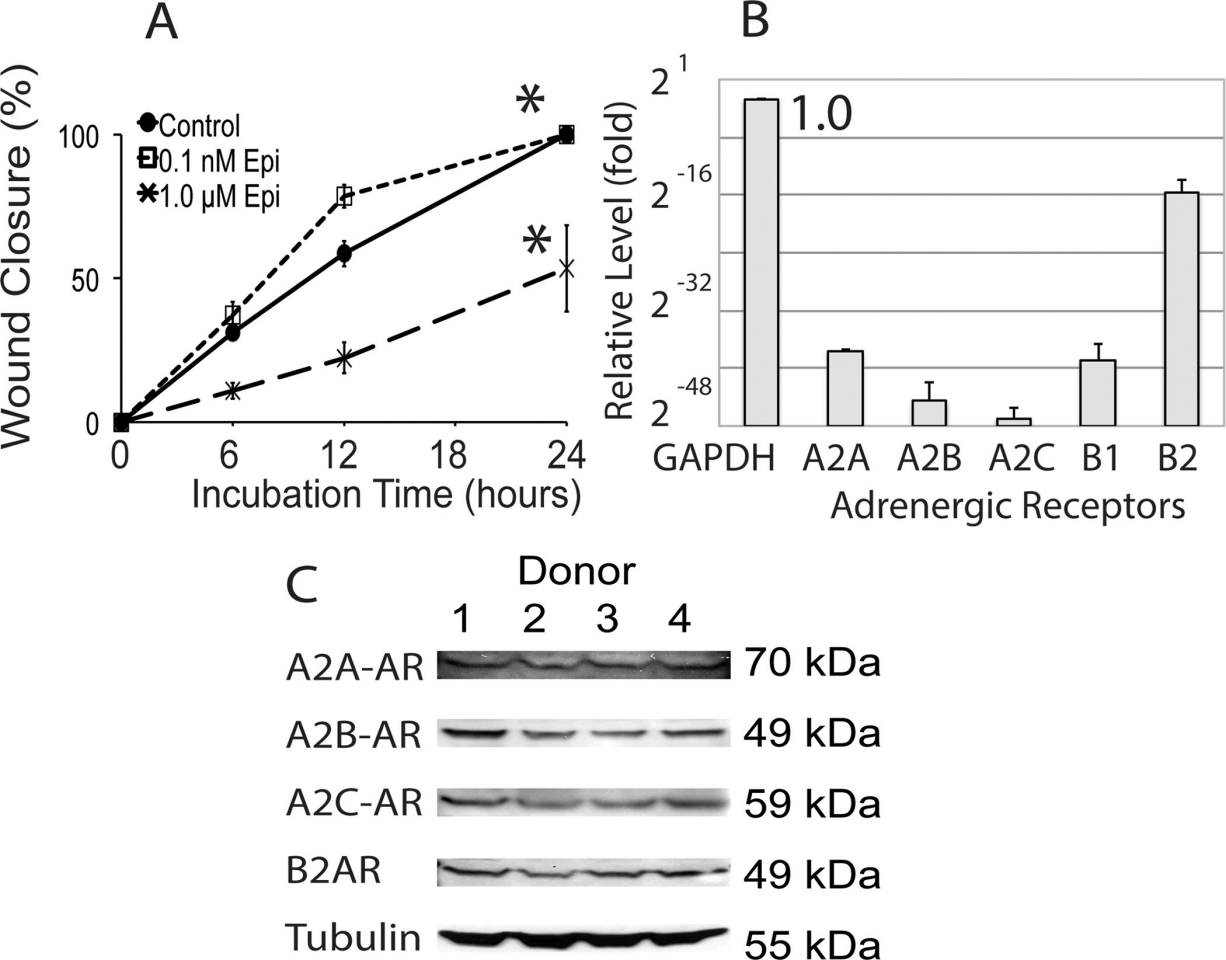
Migration patterns and expression of adrenergic receptors in human keratinocytes. (A) An increase in migration rate at 12 hours was observed in scratch assays with the keratinocytes treated with 0.1 nM (low concentration) of epinephrine compared to higher, 1 μM concentration. (B) Quantitative RT-PCR (results normalized to GAPDH expression, set as 1.0; X-axis shown in log 2 scale) and (C) Western blotting was performed to detect the expression of adrenergic receptors in keratinocytes. In addition to the B2AR expression, alpha-AR subtypes are expressed in the keratinocytes at low levels. (N = 3 keratinocyte strains, mean +/- SE, * p<0.05).

**Fig 2:**
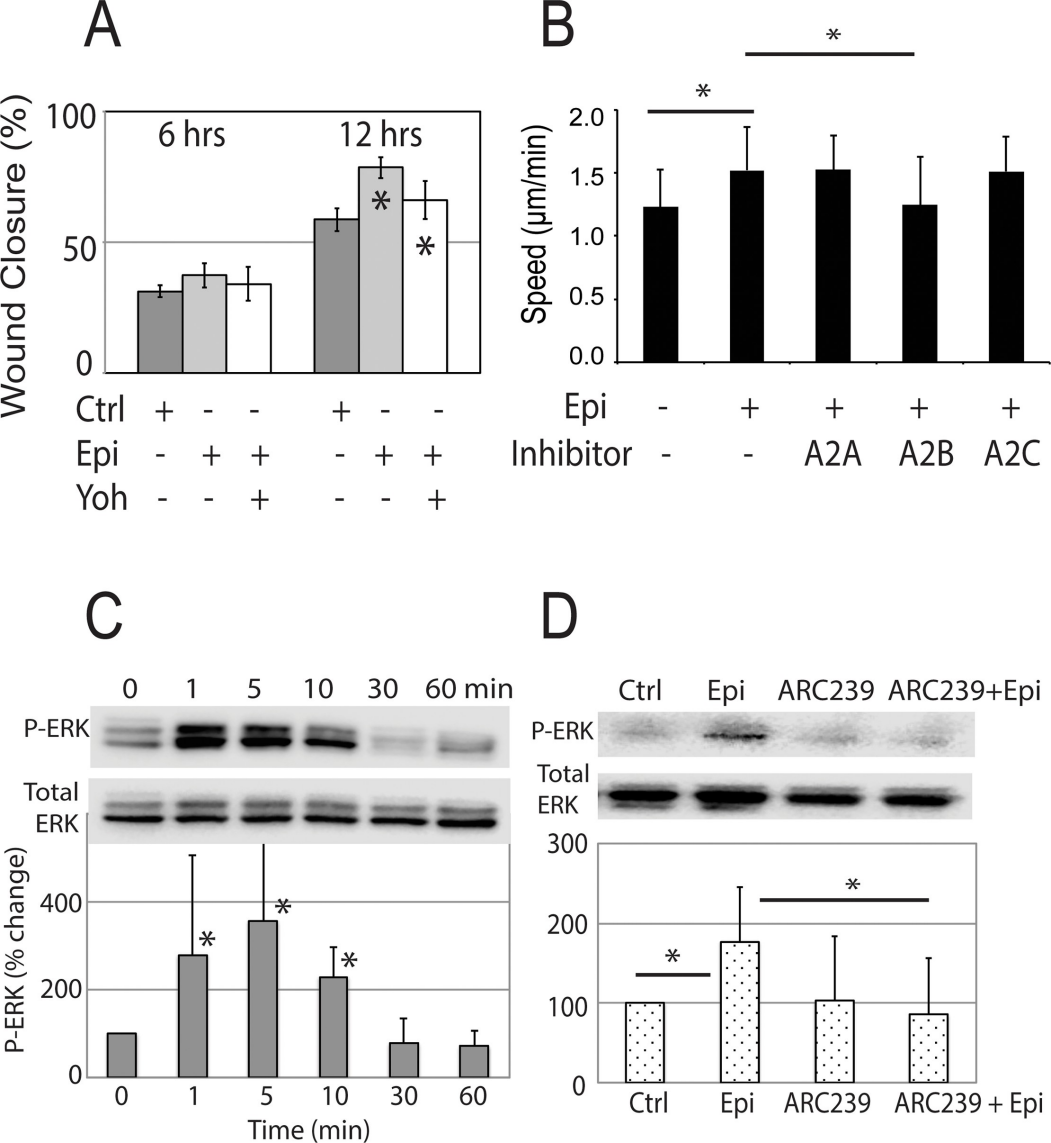
Inhibiting alpha-AR reversed the fast migration and ERK phosphorylation induced by low level of epinephrine. Epinephrine-induced, fast keratinocyte migration could be reversed by co-treatment with an alpha-AR inhibitor (A) 10 μM yohimbine (Epi + Yoh) or (B) 10 μM specific inhibitors to the A2B-AR. In (B), the individual speeds of 100–150 cells were tracked in each group in the cell migration assay. (C) Phosphorylation of ERK was increased in keratinocyte lysates at different time points after the 0.1 nM epinephrine treatment. (D) When the A2B-AR specific inhibitor was added to the epinephrine treatment, the increased P-ERK signal was abolished. (N = 3 keratinocyte strains, mean +/- SD, * p<0.05).

The neonatal human keratinocytes (NHKs) were isolated [5] from the discarded foreskin from elective circumcision and collected from de-identified neonatal male donors at UC Davis hospital in Sacramento, CA under a protocol approved by the UC Davis Institutional Review Board (IRB) Administration. Seventeen strains of NHK were screened by the single cell migration assay [23] and 3 strains that demonstrated a divergent response of significantly accelerated migration at 0.1nM epinephrine concentration were identified for this study. Cell passages between 3–6 are used. NHKs were cultured in keratinocyte growth medium (KGM, EpiLife medium, human keratinocyte growth supplements and 1x antibiotic-antimycotic, Invitrogen, Carlsbad, CA). Cells were starved overnight by cultivation in half strength supplements before the migration assays, then treated with KGM alone (control) +/- (−)-Epinephrine (+)-bitartrate salt (epinephrine, Sigma-Aldrich, St. Louis, MO), yohimbine hydrochloride (yohimbine, a non-specific alpha 2-AR inhibitor, Sigma-Aldrich), BRL 44408 maleate salt (BRL44408, an A2A-AR inhibitor, Sigma-Aldrich), ARC239 dihydrochloride (ARC239, an A2B-AR inhibitor, Tocris Bioscience, Minneapolis, MN) and rauwolscine (an A2C-AR inhibitor, Sigma-Aldrich) for 30 minutes. Time-lapse images of 50–100 cells for the single cell migration assay were captured every 5 minutes for 1 hour and migratory speeds determined as described [23]. Trend lines were added in Excel. For scratch wound assays [24], confluent keratinocytes were treated with mitomycin C (10 μg/ml, 1 hr, Calbiochem, Burlington, MA) to inhibit cell proliferation. The wound area was measured by Image J. Total RNA was isolated from cultured NHK using the RNeasy® Mini Kit (Qiagen, Valencia, CA) and reverse transcription was performed on 2 ng RNA using the High Capacity cDNA Reverse Transcription Kit (Applied Biosystems, Foster City, CA). Quantitative RT-PCR (TaqMan® Gene Expression Assays, Applied Biosystems, Hs01099503_s1 for ADRA2A, Hs00265090_s1 for ADRA2B, Hs03044628_s1 for ADRA2C, Hs02330048_s1 for ADRB1, and Hs00240532_s1 for ADRB2) was performed in duplicate on 0.056 ng cDNA in a reaction volume of 10 μl to determine AR expression in NHKs with normalization to GAPDH. The protein level of ARs and phosphorylated ERK was determined by Western blotting and 50 μg protein per lane was loaded on NuPAGE Novex 10% Bis-Tris gels (Invitrogen), transferred to Immun-Blot PVDF membranes (Invitrogen) and blocked for 1 hour at room temperature with 5% milk in tris-buffered saline (TBS). Blots were incubated with diluted primary antibodies overnight at 4°C followed by a 1-hour incubation with secondary antibodies at room temperature. Anti-A2A-AR (1: 50, Santa Cruz Biotech, Dallas, Texas), anti-A2B-AR (1: 5,000, Genex BioScience, Hayward CA), anti-A2C-AR (1: 100, Santa Cruz Biotech), anti-B2AR, (1: 2,500, Abcam, Cambridge, MA), anti-β-tubulin I, (1: 15,000, Sigma), anti P-ERK (1: 1,000), and anti ERK (1: 1,000), and HRP-conjugated secondary antibodies (1: 2,000–2,500, Cell Signaling, Danvers, MA) with enhanced chemiluminescence detection (GE Healthcare, Chicago, IL) were used to detect the target proteins. The Western blots for ARs in Fig 1C were imaged on X-ray films (Kodak, Rochester, NY), and the blots for phosphorylated ERK and total ERK in Fig 2C and 2D were imaged digitally (Odyssey, Li-Cor Bioscience, Lincoln, NE) for quantification. The time curves from the scratch assays were compared by two-way ANOVA in StatPlus and the Student T-test was used to compare the migration speeds and the intensity of Western blot bands in Fig 2.

## Results

We previously demonstrated that supra-physiologic levels (1 μM) of epinephrine inhibit keratinocyte migration and wound healing [10]. However, a divergent response is seen in some strains when keratinocytes are exposed to lower, physiologic concentrations of epinephrine (0.1 nM). Scratch wound closure by keratinocyte cultures treated with low physiologic concentrations of epinephrine (0.1 nM) was increased by 34.1% relative to control untreated cultures, but was inhibited by 62.5% when treated with high concentrations of epinephrine (1 μM) (Fig 1A, 12 hrs). The results indicate that epinephrine may differentially modulate keratinocyte migrational speed, and thus potentially alter wound closure in different individuals.

Since epinephrine can also bind to the alpha AR, we hypothesized that their activation of these receptors could explain the divergent migratory response observed at low concentrations of epinephrine. Analysis of keratinocyte cultures from 4 random donors (Fig 1B) demonstrates that the mRNA for the beta 2-AR is the most abundantly expressed AR in human neonatal keratinocytes, while other subtypes, including A2AR are expressed at lower levels. Although the alpha-1 AR has been noted in the rat and human keratinocytes and is up-regulated after burn and nerve injury, and in the patients suffering from the complex regional pain syndrome [25–27], this is the first report of expression of A2AR subtypes in normal keratinocytes. Protein expression of the AR subtypes in keratinocytes was confirmed by Western blotting (Fig 1C).

To test the hypothesis that alpha-AR activation modulates the increase in keratinocyte migratory speed observed in cultures exposed to low physiologic concentrations of epinephrine, alpha-AR activation was blocked by pre-treating the cells in Fig 2 with the non-specific alpha 2-AR inhibitor yohimbine. Yohimbine reversed the accelerated keratinocyte migration induced by the low physiologic level of epinephrine, returning cell migratory speeds to that of untreated controls in the scratch migration assays at 12 hours (Fig 2A).

To determine which specific A2AR mediates the observed migratory response, we tested whether A2AR subtype specific antagonists, BRL44408 (A2A-AR inhibitor, 10 μM), ARC239 (A2B-AR inhibitor, 10 μM) or rauwolscine (A2C-AR inhibitor, 10 μM), could alter the epinephrine-induced increased migratory response (Fig 2B). The response could only be reversed by the A2B-AR antagonist ARC239, suggesting that the signaling pathway of A2B-AR modulates the B2AR-dependent keratinocyte migratory inhibition.

B2AR-induced inhibition of keratinocyte migration requires activation of the phosphatase PP2A that subsequently de-phosphorylates the p42/44 mitogen-activated protein kinase ERK, whose phosphorylation and activation mediates migration [5, 6]. Therefore, we examined the contribution of A2AR activation to ERK 1/2 phosphorylation by co-treatment of keratinocytes with epinephrine and ARC239. We observed a rapid (1–10 min) increase in ERK phosphorylation in response to low (0.1 nM) levels of epinephrine (Fig 2C) that returned to basal level after 30 minutes. The combined treatments of epinephrine and ARC239 reduced the ERK phosphorylation induced by low (0.1 nM) epinephrine concentrations (Fig 2D), suggesting that A2B-AR modulates B2AR signaling. Inhibiting A2B-AR activity antagonizes the B2AR-mediated ERK phosphorylation, resulting in the observed change cell migration speed.

Thus, in contrast to the decreased migration response of keratinocytes to stress-associated, supra-physiologic levels of epinephrine, here we demonstrate that a subset of strains exhibit a divergent response to low physiologic concentrations of epinephrine, with up-regulation of ERK 1/2 phosphorylation that is reversible by an A2B-AR inhibitor.

## Discussion

The B2AR is highly expressed in undifferentiated, migrating keratinocytes [21, 28] in the basal layer of epidermis, and these cells can also generate catecholamine (epinephrine) ligands for the receptor [5, 19, 22]. Keratinocyte migration plays a critical role in wound re-surfacing [29, 30]. Therefore, the modulation of B2AR signaling in keratinocyte migration can play an important role in controlling the rate of wound healing.

Here we show that the B2AR signaling paradigm can be modulated by alpha adrenergic receptors, also expressed in the epidermis. We note that the adrenergic antagonists are not completely selective, and gene silencing could be a better approach to test the hypothesis. However, due to the inefficiency of depleting receptors in primary keratinocytes (for example only 50% knock down of expression of the epithelial sodium channel [31]) and a very limited generation time in cultured primary keratinocytes, even with knock down, it would be difficult to demonstrate convincingly that other receptors are not involved. Therefore, for this study we chose to use the more tractable pharmaceutical approach. Possible crosstalk between the alpha and beta ARs is observed in mouse cardiac and embryonic fibroblasts. The scaffold protein, arrestin, mediates the signaling between the 2 receptors and increases the phosphorylation of ERK possibly via a G-protein-independent signaling events [32, 33], suggesting that a combination of the drug treatments targeting the alpha and beta adrenergic receptors may compound or diminish the signaling and the efficacy of each drug on cardiac remodeling. Our results indicate that a subset of patients will have divergent responses to epinephrine in their skin wounds, and indeed, similar observations have been made in different clinical scenarios. For example, a small subset (16–19%) of patients receiving epinephrine for food-induced allergic anaphylaxis [34, 35] require multiple doses, suggesting that these individuals are not responsive to the initially administered low dose of epinephrine [35]. The expression levels of each of AR subtype, or their polymorphisms, as well as receptor internalization rates likely differ in each individual, which may account for the observed divergent responses.

BAR antagonists (beta blockers) are increasingly used topically to treat skin diseases. The non-specific antagonists for beta AR such as propranolol and timolol, are widely used for treating infantile hemangiomas [36–39]. Topical application of timolol is used to treat a host of dermatologic diseases [40, 41], including epidermolysis bullosa [42], Kaposi sarcoma [43, 44], acne and acne rosacea [45], and improving scar outcomes [46]. Timolol has also been found to be effective in treating chronic wounds [14–17]. However, this beta blockade improves healing in some, but not all, patients with chronic wounds [42, 47, 48]. Thus, the cell migration assay reported here could provide an *in vitro* tool to examine patients who may have the divergent reactions to epinephrine, and to determine the therapeutic strategy of epinephrine-derived treatments.

There are some limitations to this study. The keratinocytes used are derived from neonatal foreskin, due to the better replicative capacity of neonatal tissues. Thus, results are limited to cultured, rather than *in vivo* keratinocytes. Sexual dimorphism has been reported for B2AR responses [49–51], so confirming the observed results in keratinocytes derived from females will be important. The de-identified neonatal foreskins collected for the study do not provide any information regarding potential racial differences in responses, and this could be a factor to investigate especially in view of racial B2AR polymorphism differences [52–55].

**Fig 3:**
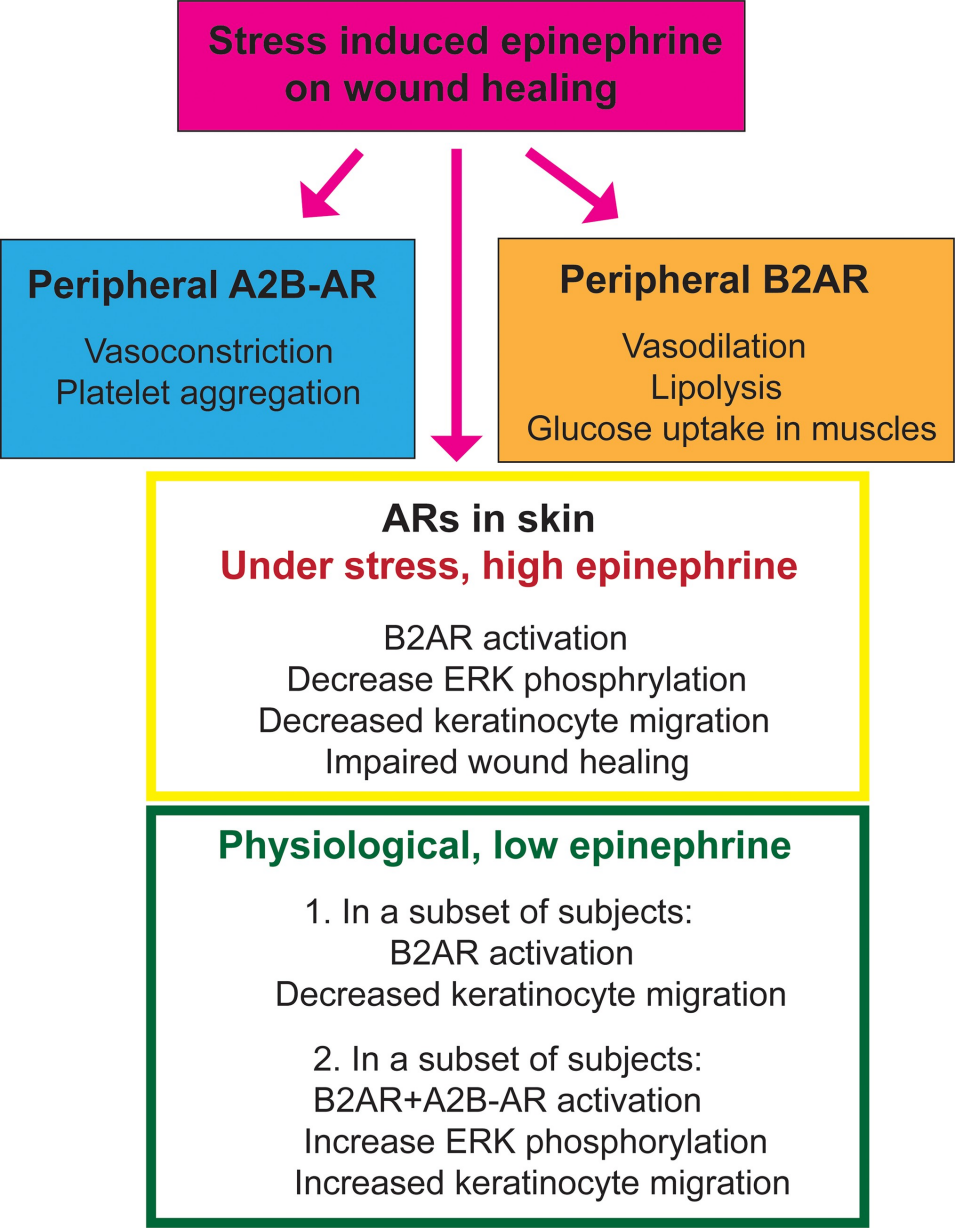
A summary of functions of peripheral A2B-AR and B2AR in wound healing.

Taken together, the present study suggests an interaction between A2AR and B2AR in a subset of human keratinocytes, which influences the epinephrine-mediated keratinocyte migration. A summary of the peripheral functions of A2B-AR and B2AR in wound healing is shown in Fig 3. Future studies will investigate whether a combination of an A2B-AR agonist and B2AR antagonist can promote keratinocyte migration and improve wound healing.

## Supporting information

S1 Fig Raw images of the original Western blots. The original blots for Fig 1C, 2C and 2D.
(ZIP)
