# Supplementary figures and images for: Alpha and beta adrenergic receptors modulate keratinocyte migration
Source: PLoS One. 2021 Jul 2;16(7):e0253139. doi: 10.1371/journal.pone.0253139 (PMC8253387; doi:10.1371/journal.pone.0253139)

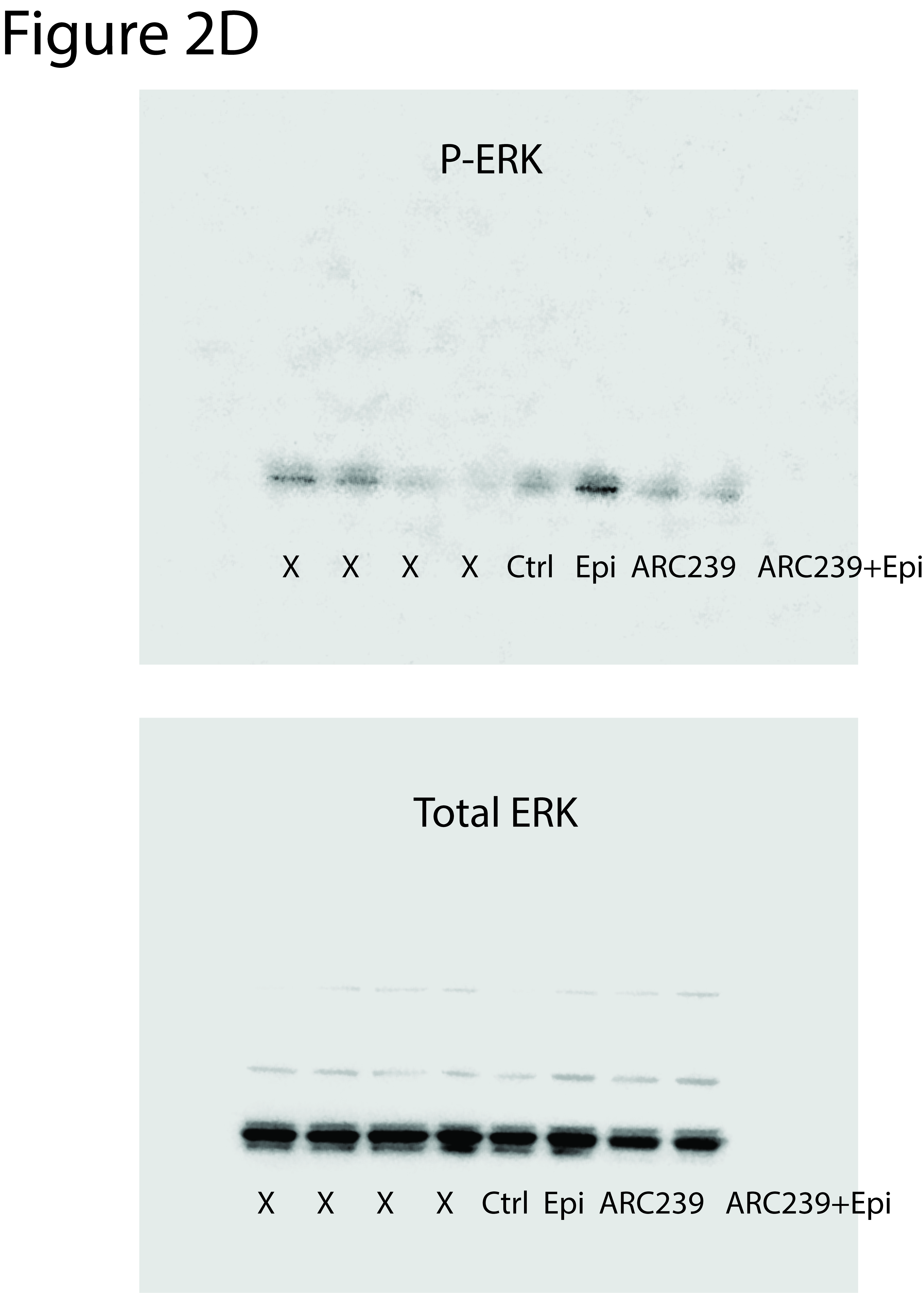

Supplement: S1 Fig — The original blots for Fig 1C, 2C and 2D. (ZIP) [file pone.0253139.s001.zip › New Figure S1-original blots/page 4-original Fig 2D.tif]

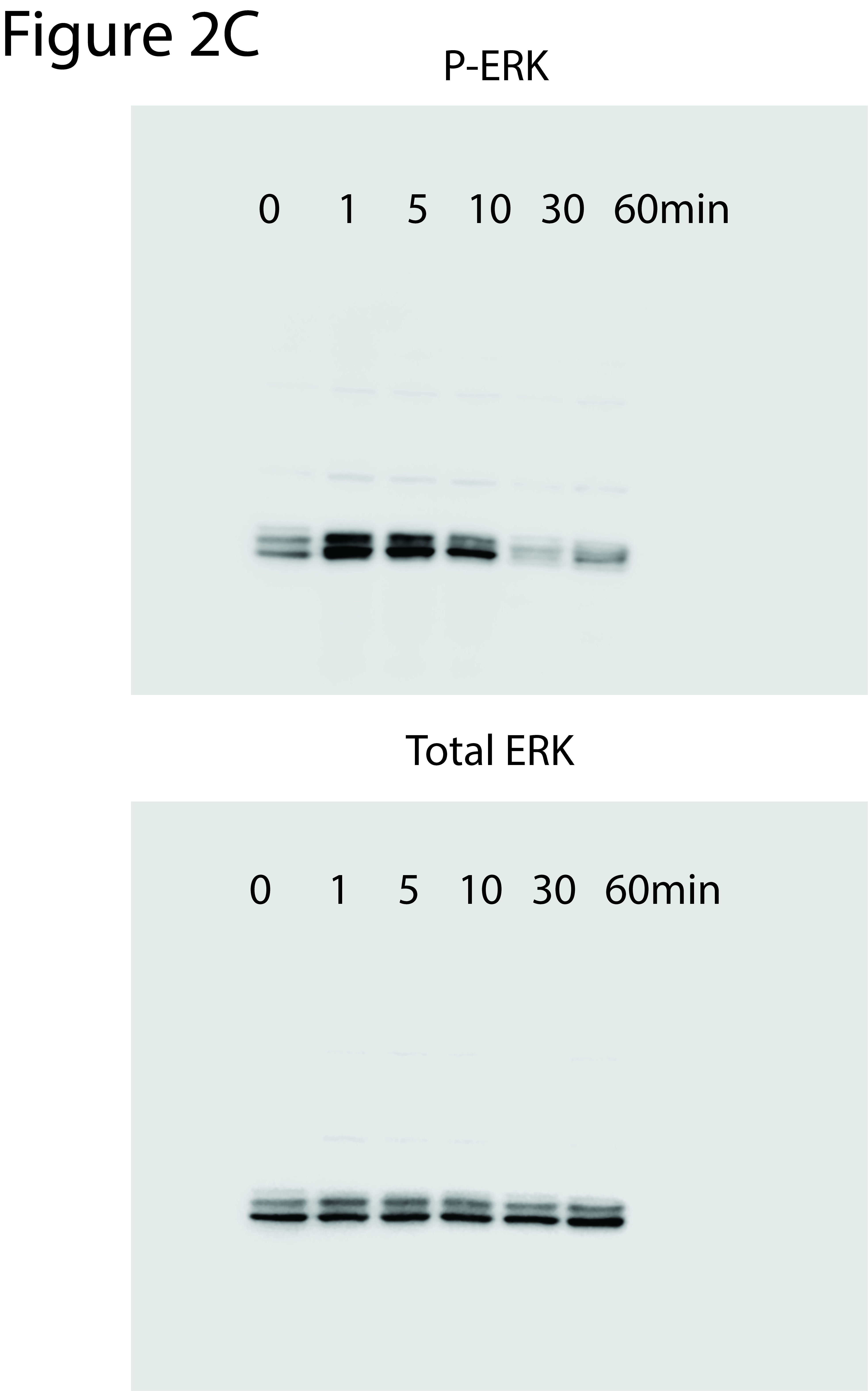

Supplement: S1 Fig — The original blots for Fig 1C, 2C and 2D. (ZIP) [file pone.0253139.s001.zip › New Figure S1-original blots/page 3-original Fig 2C.tif]

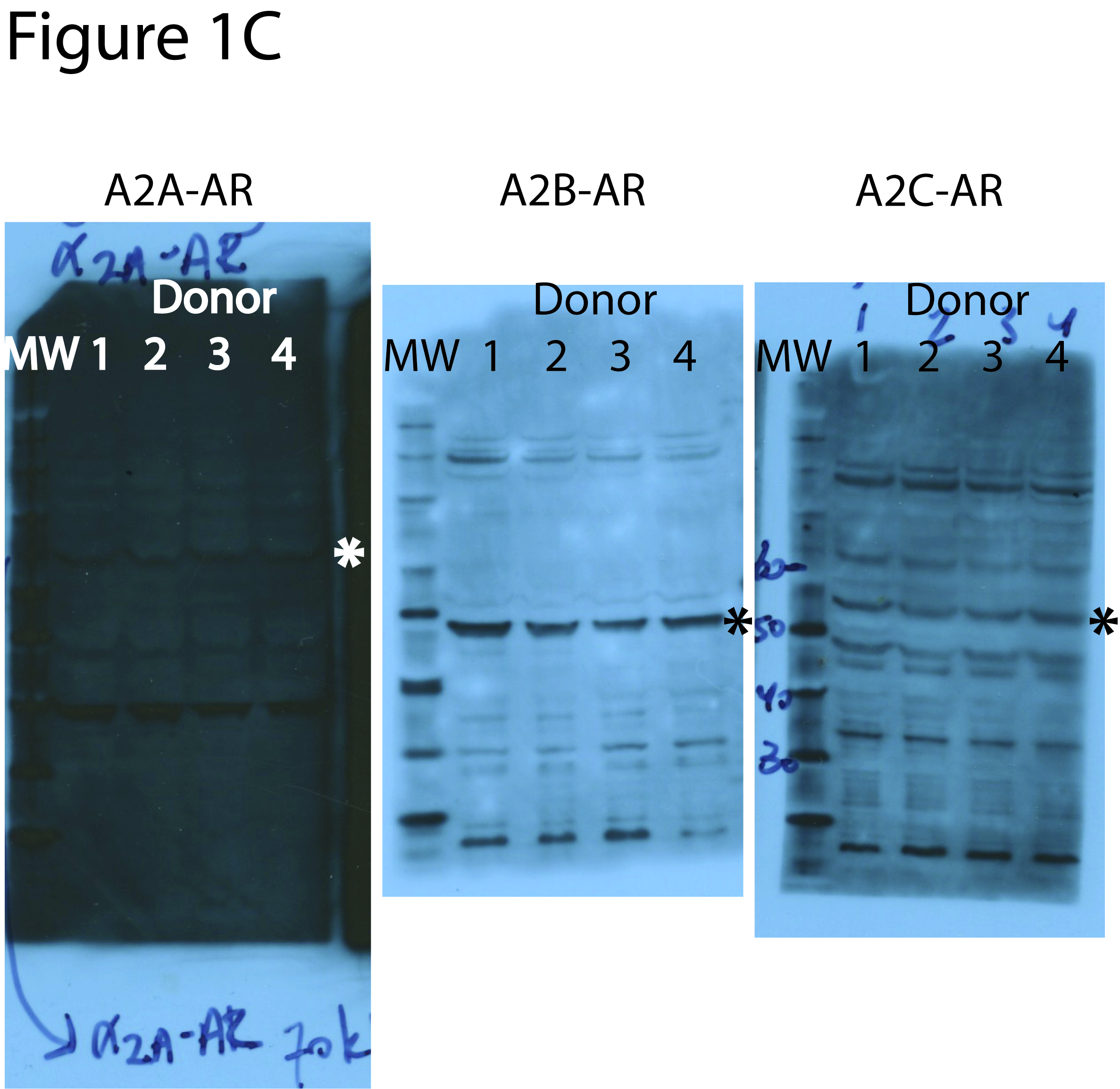

Supplement: S1 Fig — The original blots for Fig 1C, 2C and 2D. (ZIP) [file pone.0253139.s001.zip › New Figure S1-original blots/page 1-original Fig 1C-part 1.tif]

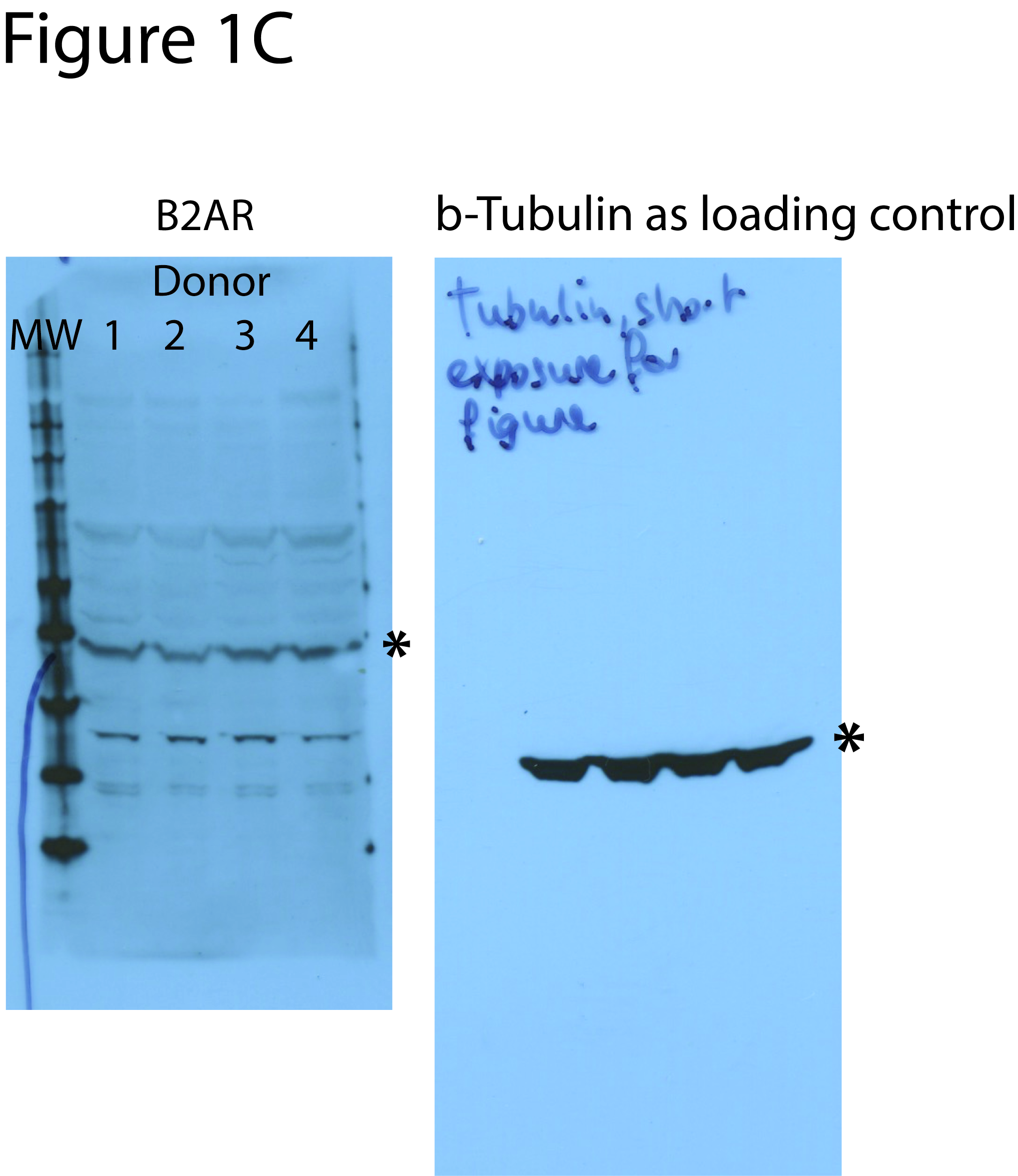

Supplement: S1 Fig — The original blots for Fig 1C, 2C and 2D. (ZIP) [file pone.0253139.s001.zip › New Figure S1-original blots/page 2-original Fig 1C-part 2.tif]
